# Experience of conductive hearing loss and impacts of hearing aid use throughout life

**DOI:** 10.3389/fresc.2024.1491473

**Published:** 2024-12-11

**Authors:** Thomas Hampton, Manuel Loureiro, Kevin Mortimer, Deborah Nyirenda

**Affiliations:** ^1^ Department of Clinical Sciences, Liverpool School of Tropical Medicine, Liverpool, United Kingdom; ^2^ Faculty of Health and Life Sciences, University of Liverpool, Liverpool, United Kingdom; ^3^ Audiology Department, University Hospitals Sussex NHS Foundation Trust, Brighton, United Kingdom; ^4^ Malawi-Liverpool-Wellcome Trust Clinical Research Programme, Blantyre, Malawi; ^5^ Cambridge Africa, University of Cambridge, Cambridge, United Kingdom; ^6^ Department of Paediatrics and Child Health, School of Clinical Medicine, College of Health Sciences, University of KwaZulu Natal, Durban, South Africa; ^7^ Department of Respiratory Medicine, Liverpool University Hospitals NHS Foundation Trust, Liverpool, United Kingdom

**Keywords:** deafness, hearing, hearing loss, quality of life, social norms, masculinity, qualitative, technology—assistive/supportive

## Abstract

**Introduction:**

Hearing loss and Deafness/deafness affects as much as 5% of the world's population and has a considerable health and economic burden. We explored the relationship with hearing and hearing aids as well as other assistive technology for health in general with a cohort of UK adults who have conductive hearing loss. We anticipated that insights could lead to greater understanding for the delivery of assistive technology (AT) for conductive hearing loss and the participant's lived experience related to technology and society.

**Methods:**

This study presents the qualitative findings from a mixed methods study exploring the story of each participant's hearing, the impact on their lives and their experience and use of AT. A purposive sample of 33 adults with conductive hearing loss took part in semi-structured interviews. Participants were aged ≥18 years and had previously attended outpatient ENT or audiology clinic at University Hospitals Sussex NHS Foundation Trust. Transcripts underwent thematic analysis.

**Results:**

The overarching theme was “A changing relationship over time with deafness, themselves and society”. The three principle sub-themes of the interviews were “a technological world” describing the necessity of interaction with people & technology' both as children and adults, then the concept of “Normalised Marginalisation”-the struggle of childhood and school in the face of social norms' and typicality. Finally, there were issues raised about visibility and “the visible display of D/deafness”, tied to aesthetics, vanity and traditional ideas about masculinity. Many participants described their adoption of new technology or devices as “*transforming their life*” and their quality of life without assistive technology as significantly impaired.

**Conclusion:**

Insights from this study described the experiences of adults with conductive hearing loss and the ways in which they have a difficult relationship with their deafness, including how they felt and viewed themselves and how they interacted with society, particularly as children and young adults. The potential for benefit of assistive technology for hearing health was deemed by many participants to be a necessary bridge integrating them in relationships with other people in society. Early notions of disability, typicality and social norms frequently persisted into adulthood and these insights should be considered by all those professional seeking to provided hearing health assistance to individuals with conductive hearing loss.

## Introduction

Hearing loss affects 1.5 billion people globally with over 5% of the world's people requiring some degree of rehabilitation for a disabling hearing loss (1). Data from the English Longitudinal Study of Ageing suggests 48% of people age 50 years and over have some degree of hearing loss (2). Health economic analysis suggests that £25 billion a year is lost in productivity and unemployment alone (3).

Hearing loss can be conductive, sensorineural or mixed but this study focuses on conductive loss which has a prevalence in 2016 estimated at 704,000 people in the UK or around 8% (4). Conductive deafness refers to tympanic membrane (ear drum) or ossicle (ear bone) issues rather than signal issues with the nerves or the brain's interpretation of sound. Otitis Media is a prevalent cause of (usually) conductive school age hearing loss (5). Individual studies have found conductive hearing loss in 8% of schoolchildren in Iran (6) whilst a worldwide systematic review found ranges from 0.4% to 64.5% in new-born hearing screens (7). Tympanic membrane perforation alone can cause conductive hearing loss and reported prevalence ranges have included 1.3% in China (8), 2.1% in the USA (9) and 3% in South Africa (10).

Hearing loss experiences and outcomes can be heterogeneous and varied (11). Rather than using the phrase hearing impairment to describe levels of hearing threshold loss, in the UK the phrase D/deaf is often used (12). “Lowercase d” deaf refers to all levels of deafness and can be used to describe anyone with any degree of loss. This is a conscious attempt to move from the medical model of disability, that perceives hearing loss primarily as a condition or impairment that needs to be treated or cured, phrases which are felt to marginalise some individuals (12).

“Uppercase D” Deaf is a sociocultural phrase generally encompassing individuals who use sign language [e.g., British Sign Language (BSL) in the UK] as their first language. In general, these individuals are said to identify as part of a Deaf community with a distinct cultural identity and linguistic heritage and a longstanding struggle for recognition of this culture, language and rights in a society that largely prioritises spoken and auditory communication. Deaf individuals usually highlight a social model of disability that seeks greater acceptance, adaptation and integration rather than a goal of medical correction (13).

Previous research has attempted to negotiate the “uneasy positioning of D/deafness and disability” and it should not be assumed that they are synonymous (14). The experience and identification of D/deafness is tied to self-perception as well as the consequences of discrimination from an otherwise hidden nature of deafness (14, 15) which can place particular emphasis on thoughts feelings and decisions around “disclosure” (16).

Because the visual cue of assistive technology or hearing aids essentially identifies individuals as “different” or “not normal” (17), this has been suggested as a reason why many with D/deafness either deny or conceal their hearing loss initially and then postpone seeking assistance (11). Many who could benefit from hearing aids (18) do not wear them and concerns about this visual identification exacerbating stigma has led to discussion about total or near complete concealment of aids, including one study about theoretically “invisible” cochlear implants (11). It is well documented that for individual patient reasons (principally reported as difficulty with dexterity or comfort, in addition to concerns regarding stigma and appearance) 20% of adults currently do not use their hearing aids at all and the uptake of assistive technology for hearing is as low as 20%–30% of individuals who could potentially benefit from intervention (18). In 2007, in Great Britain, 12% of 31,793 respondents aged 55–74 years reported hearing loss (19). Only 3% were currently receiving an intervention, a figure which correlates with estimates for those living in low income settings a decade later (20). The participants principally lost ability to hear speech in noise, and overall the median duration of symptoms was around 6 years (19). More recent international estimates have only improved to 62% of the patients prescribed hearing aids wearing them (21).

As part of a larger trial assessing acceptability of over–the-counter, non-audiologist fitted bone conduction devices, we conducted exploratory interviews in a cohort of purposively sampled adults in Brighton UK with known conductive hearing loss. In addition to soliciting opinions of the performance of the new devices we wanted to assess the impact of the participants hearing loss as children and as adults and the benefit that assistive technology for hearing health (hearing aids or otherwise) had on their life.

We anticipate insights from this study will lead directly to future study design and potentially to device development in response to current trends in hearing aid use amongst those who have capacity to benefit. We also hope these insights can be targeted explicitly at delivering assistive technology for conductive hearing loss.

## Methods

### Design

This study used semi-structured interviews with individuals who had conductive or mixed deafness with prior experience of using AT (to rehabilitate their conductive deafness). The interview questions were not fixed and the conversation was allowed to flow in the hope that this may allow participants the flexibility to discuss D/deaf concepts and hearing aid experiences which the researcher might not have anticipated (14, 22–24). The interviewer was not D/deaf and this should be considered as a potential cause of researcher-participant hierarchy and an impairment to potential reciprocal conversation (17) but the focus remained on the participants own perceptions of device design and any concerns or stigma from their viewpoint and experience.

### Participants

A combination of purposive and convenience sampling sought eligible adults age ≥18 years who have previously attended outpatient ENT or audiology clinic at University Hospitals Sussex NHS Foundation Trust (UHS). All participants required documented conductive hearing loss regardless of aetiology (unless otherwise indicated in exclusion criteria). Participants were included who had previously used any audiological device (hearing aids, assistive technology, bone conduction devices or surgical auditory implants to treat a conductive loss). All participants had to be able to converse in English (English did not need to be first language- but participant-determined fluency was the criteria).

Children, adults with cognitive impairment that would limit device engagement, and those with medical conditions that would preclude wearing the hearing device from the main quantitative study (i.e., severe dermatology) were all excluded but this threshold was self-determined by participants and their family (there were no exclusions amongst attendees). In total, 167 participants were contacted by letter to solicit interest in the study. A target of 30 total participants was initially sought. Thirty-six patients expressed interest and thirty-three participants attended and completed all the consent process and the in-person elements of the study.

All of the participants had some experience using other assistive technology for hearing and opinions were sought in reference to all devices, not just bone conduction devices. Given that the purpose of the qualitative interviews is to contextualise responses and understand the impact of hearing loss in this population, we felt that a sample of this size would give us a reasonably representative insight from our participants which could be extrapolated to experiences from similar cohorts. Previous literature suggests that data saturation for novel codes in heterogeneous populations can be achieved in approximately 30 interviews (25). Despite due consideration for choosing participant numbers, formal data saturation was not sought or measured in this study based on our epistemological standpoint (26).

Sample demographics about the interviewed participants are available in Appendix 1.

Table A1 reports demographic data including levels of hearing loss divided into severity cohorts. The participant postcodes were also assessed to determine surrogate information about our participants (basic demographic data is also provided in results). Postcodes were assessed against the UK government's English indices of deprivation 2019 data on Lower-layer Super Output Area. This data offers an Index of multiple deprivation (IMD) rank. Our participant cohort comprised the full range of IMD deciles from 1 to 10 which should indicate that the opinions and experiences of a wide range of participants was included although the cohort had a median IMD decile rank of 7.1 (meaning that the participants in this study were living in neighbourhoods that are slightly less representative of deprived UK communities, even if this doesn't reflect their own personal circumstances).

### Procedures

The 33 participants (aged 21–82 years, mean age = 66 years, SD 16) agreed to take part in semi-structured interviews to share their experiences of using hearing aids and living with conductive deafness. Although earlier parts of the interview focused on perceptions and impressions of a new hearing device, analysis here focused on the biographical and experiential comments from participants as we explored their relationship with hearing and hearing aids as well as other assistive technology for health in general.

An initial pilot interview with another hearing health professional outside the authorship was used to adjust the prompts in the topic guide before finalising. The interview schedule (which includes some questions not addressed here, which were directed towards the evaluation of a new bone conduction device) is available in Appendix 1).

Interviews occurred in an audiology clinic room after confirmatory hearing and speech testing at the study site in the seaside town of Brighton, UK, in May and June 2022. An information sheet describing the purpose of the study and the interview, as well as the roles and aims of the researchers involved was provided via email and then offered again in person on the day of participation. Participants completed written consent before their interview. All invited participants expressed willingness to participate after hearing tests. Interviews occurred in person but a laptop using Zoom software (Zoom Video Communications) and Philips 3200 SpeechMike Pro USB Microphone (LFH3200) was used to create an audio recording of the interview which was saved as anonymous audio files, and mapped to study ID. Covid-19 precautions followed NHS Hospital Trust Policy at the time of interviews. Participants were offered £20 as compensation for their time and contribution towards their transport costs from across the south coast to the study centre.

The interviewer was a male doctor (TH) and both interviewees and interviewer conducted interviews seated, with some participants preferring to allow a partner/guardian or family member to sit-in depending on personal preference. The overall research time for participants including the quantitative elements came to over an hour so a shorter interview target duration was set so that no interview would exceed 20 min. Anonymised handwritten field notes were taken relating to phrasing and reception of questions, as well as any additional comments made during the potentially more natural discussions “off-tape” with participants permission only. The semi-structured interview schedule was used flexibly; where topics were previously covered, prompts were omitted, adapted or expanded on, depending on each interaction.

### Analysis

A thematic analysis of the dataset was conducted following the six-phase methodology described by Braun and Clarke (27). Thematic analysis (TA) was chosen as a flexible approach that is suited to the research question in this context, used in the context of metaparadigm approaches to D/deaf epistemology (28) (in this case: essentialism (some objects have intrinsic properties) (29) and constructivism (30) (the idea that knowledge is constructed rather than just passively taken in, using people's experience of the world and their reflections on what they understand as pre-existing knowledge. Although parts of this study took an empirical approach, we can acknowledge the positionality of the lead researcher (TH) who has undergone quantitative, positivistic and realist training and therefore we have adopted a “small q” pragmatic TA approach. The interviews and data from this qualitative study will form part of a pragmatic mixed methods approach, nested as a discrete explanatory sequential design with the quantitative elements of a study with the same participants) rather than the “Big Q” reflexive TA advocated by Braun and Clarke recently (31). Thematic analysis in this study was inductive with no *a priori* coding framework. Therefore, during the analysis, each interviewee's descriptions and experiences of events and their perception of the meanings behind them, were assessed and concepts developed from the data rather than adjusting responses to fulfil or refute existing overarching theories. Although the researcher team has had prior exposure to studies and personal accounts of the impact of D/deaf and hard of hearing participants, the analysis did not seek to confirm or allocate codes or themes to these expected experiences if they were not apparent.

Participants' study IDs are provided below alongside quotes to indicate the variety of participants contributing to overall themes. As per the original study protocol, participants who requested feedback will receive email or postal feedback on the findings of the study after submission to a scientific journal so that they can appreciate the scientific knowledge they helped create. Triangulation and Member checking or informant feedback to assess descriptive, interpretive and theoretical validity was not conducted (32).

Audio recordings were transcribed using a professional transcription service (https://www.uktranscription.com) and coded using NVivo V.12 Pro software (QSR International).

As per recommended thematic analysis processes, transcripts were read from start to finish to familiarise with the content, then re-read to develop common topic “codes”. Each transcript was then re-read and codes were combined into categories. Categories were larger groupings related to the same topic i.e., comments about issues with batteries running out or charging devices needing a socket whilst out on the go, might reasonably be grouped into a “power and reliability” category then these categories combined under unifying themes until no further themes were identified. After initial coding by TH, all members of the research team individually discussed the identified and recurring themes until a broad understanding was achieved. No explicit consensus was sought and TH made final decisions about theme refinement with further contextual and interpretive insights and supervision provided by DN. An iterative approach was adopted for the thematic phases, as data acquisition from the final interviews took place weeks after the author had time to reflect and consider the first interviews, even though there was no analysis of early transcripts formally taking place. The final stage of analysis involved determination and synthesis of underlying narratives within the interviews relating to impact of conductive deafness and hearing aid use.

### Patient and public involvement

Patients and members of the public were not involved in the design of this study although feedback from pilot studies was incorporated in the interview prompts and final data analysis. Patients were integral to the conduct of the research itself advising on dissemination and offering impactful insights and lived experiences. Both patients and public will benefit from planned dissemination of this research including Public Communication of science events such as Pint of Science (Public audiences for research dissemination talks as part of a wider National Lottery Heritage-funded project) and World Hearing Day Events. Copies of published articles will be sent to consenting participants directly as requested.

These study methods and reporting followed Consolidated Criteria for Reporting Qualitative research guidelines (33).

### Ethics approval

This study involved human participants. Ethical approval and sponsorship were obtained from the Liverpool School of Tropical Medicine (21-079) and the national NHS Health Research Authority Research Ethics Committee review was sought and approved (IRAS 304976). The study was also registered with the UHS Clinical Audit and Research Department. Participants gave informed consent to participate in the study before taking part.

The study was adopted as an NIHR Portfolio study and assistance provided by local CRN team.

## Results

An overarching theme and three subthemes were developed from these interviews. “A changing relationship over time with deafness, myself and society” was the overarching theme of these interviews. The three principle sub-themes of the interviews were: a technological world”, “Normalised Marginalisation” and “the Visible Display of D/deafness”.

### A changing relationship over time with deafness, myself and society

Many participants described their adoption of new technology or devices as “*transforming their life*” and their quality of life without the aids as significantly impaired. Participants explained the necessity of interaction with people & technology affiliated with deafness, both as children and adults. Participants spoke about “othering” and marginalisation particularly during childhood and school in the face of social norms and typicality. Participants thoughts and feelings about appearance and visibility in particular often prompted story sharing about prior experiences. Many alluded to their perceptions about personal appearance and stereotyped normal appearance, with exceptions intrinsically linked to disability. The metaphorical threshold over which participants had to step was largely a visual announcement of D/deafness.

Standing out visually appeared to endorse perceptions ingrained in society, and many participants reflected on how they preferred to live without engaging this.

Participant 16 (68 years old male): ‘… the device … there’s no way I would have worn that’

Definitive responses like this about the appearance of assistive technology were both positive and negative. Some participants were happy with a design of hearing device which could mask their disability—if assistive technology looked like a music device, it came with no associated “negative” connotations.

Participant 2 (70 years old female): ‘People would wear them because they look like normal headphones… My cousin has some of these that he just uses for music!’

Music and listening or hearing for pleasure (not necessity) appeared to be an act of normality or joy that resonated positively in this cohort against a backdrop of difficulty hearing in their lived experience.

Participant 4 (73 years old female): ‘…looked cool, and you wouldn’t be embarrassed using them, because a lot of people go around with earphones anyway’

For many participants, we interpreted these comments as reflections on “normality” and disability.

Participant 5 (47 years old female): ‘all the youngsters wear headphones these days…so nobody would be aware that you’ve actually got hearing aids’

Interviews highlighted the hidden nature of deafness, and the difficulty experienced by participants who considered themselves disabled, of coping with a hidden disability in an intolerant and ableist society. Many reflected on personal shame or stigma, often recalling significant issues during childhood or school.

Most participants had experience with multiple devices, constantly being updated as technology improved.

Participant 20 (68 years old male): ‘right from the beginning 20 odd years ago, when I had my first hearing aid…as I was finding things getting worse, the next stage came along. I mean this one I’ve only had 3 years’

The overarching theme was that each participant had been on a personal journey and developed a number of relationships during their lives, which were marked chronologically by the passing of time, the changing of devices but also by the impacts of experiences. These relationships and experiences related to interacting with others and changing perceptions of themselves, including their self-acceptance, and the development of their own tacit knowledge and interactions or openness to technology. This also reflected the impacts of progress and adaptability, individually and in society at large. Hence, “a changing relationship over time with deafness, myself and society”.

### “A technological world-the necessity of interaction with people & technology”

Participants described the impact of their hearing on their ability to communicate and interact with partners, friends and work colleagues. Some participants clearly felt reliant on their regular device:

Participant 12 (74years old male): “If I didn’t have my hearing aids then I’d be lost completely”

Some participants also felt their hearing caused difficulty in their orientation and positioning, as well as balance and confidence. This included scenarios like crossing the road, or the more complicated steps involved when catching a train from a station platform. Participants felt their hearing issues significantly altered how they interact with technology, such as mobile phones and televisions (TV). For many, TV or phone issues were a major cause of stress and annoyance, personally and to the people around them, leading to a loss of confidence, misunderstandings with tradespeople or arguments with friends and family. Many participants said that in addition to their current hearing aids, they were reliant on speaker phones and other assistive technology and one described needing a flashing light as a surrogate for a functioning doorbell as she couldn't hear the bell.

Participant 26 (82 years old male) ‘…I struggle to hear mobile phones, I have to have it on speakerphone and then they’re still not loud enough sometimes’.

Many participants described the necessity of elaborate physical and verbal coping strategies (variously described as “compensations”, “get outs” or “getting around things”) for mitigating their level of hearing loss, particularly when socialising or in work meetings.

Participant 20 (68 years old male) “…if I didn't sit on the left-hand side as it were with the chairman down on my left… and people this side on my better ear… I'd have problems”

Strategies discussed varied from getting to meetings early to choosing the least acoustically hostile seat in a room, sitting in stereotyped positions (always with the good ear to the meeting-chair, or close to the front, near the speaker), and repeatedly using stock responses, aggression or jokes to deflect from miscommunication.

Nearly all participants shared stories of behaviour change and difficulty experienced when coping with a hidden impairment or disability in the structures of society built for typicality. This included seeking solace in job roles or hobbies where communication was unexpected or unrequired, or withdrawing from wider society. Many reflected on personal shame or stigma, including “personal hang-ups about disability” which some directly attributed to traumatic hearing-focused childhood events:

Participant 16 (68 years old male) ‘I probably just didn’t want my peers to know anything about me which was, as it were, disabling’

### Normalised marginalisation-the struggle of childhood and school in the face of social norms

Although it was not a universal collective experience in the participants, some participants, particularly those older than 60 years, tended to describe more severe early school experiences. These were in the context of minimalist health seeking behaviours that were culturally normalised in their families and society at those times.

Participant 14 (82years old male) ‘…only when I was working, in my teens, and into adult life, that I realised I was no more stupid than anyone else’

Difficulty finding acceptance or integration as a result of communication difficulties among peers was reported as a school experience by most participants.

Participant 33 (82 years old female): ‘we said rat and you said cat…they used to giggle. I used to giggle at first but after that I got embarrassed’

But participants of all ages, even under the age of 40 years, remembered the way they were treated with hearing loss in school, even in recent years, with bullying and some persistent stereotypes, including trying to ignore the problem.

Participant 27 (25 years old male): ‘I…didn’t do anything about it until I was much, much older’

Memorable friendships that looked beyond disability at school and then again in later life were often mentioned.

Participant 09 (32 years old female): ‘[my hearing loss] was the bane of my life…. I think sometimes you portray being a bit slow because you misunderstand…’

There was a realisation as adults that these early social interactions probably impacted the participants personalities, outlook and engagement with society far more than just their grades or performance in school work:

Participant 14 (82 years old male): ‘my parents were told by the teachers that I was slow and backward. And that went on for some years, until I was about 10 or 11, and [then] they realised I was actually deaf….but the labels are firmly stuck on you then….[so] I left school at 15- when you’re told you’re slow and backward by everybody, you think you are’.

Participant 21 (73 years old female): ‘I was in primary school, and I just suddenly couldn’t hear very well. I got a few comments, people thought I was being ignorant, they thought I was just being rude’

Participants described widespread impacts of biases or preconceptions about deafness on their thinking, and self-perception, with lower achievement or dismissive attitudes leaving imprints on their identity and essentially how they valued themselves in society, in a way that persisted through their whole life.

Participant 34 (65 years old female): ‘people think because you’re deaf, they think you’re daft’.

Participant 27 (25 years old male): ‘I know how I struggle and I know how it impacts on my life and if it had been picked up many, many years earlier than it was, my life would have been a lot different.’

### The Visible Display of D/deafness: a battle of vanity, masculinity and acceptable aesthetics

Participants of all genders spoke about appearance and aesthetics as related to both the new device from the quantitative element of the study and their long-term use of other hearing devices in the past.

Participant 10 (61 years old woman): ‘I’m a vain person to an extent. I am self-conscious of the fact that I have a bone-anchored hearing aid’

For some participants having an obvious hearing aid of any description was something they found troubling given prior experiences. Many participants said they were more comfortable with devices as they grew older, reflecting perhaps that changes from their initial objections (which at the time, most put down to aesthetics) might represent their growing personal acceptance of their hearing, or potentially a growing self-confidence and embrace of identity.

Participant: 35 (77 years old female) ‘…I think I probably was, a lot of the time, in denial of my deafness’

But others never managed to change their thinking even in later life:

Participant 09 (32 years old female): ‘because I had a BAHA [bone anchored hearing aid] on a headband, when I went to secondary school…and people bullied me badly for that. So…it sort of put…a resentment against me wearing things like that…’

Underlying thoughts and feelings about these cosmetic preferences were varied. Many participants talked about their personal perceptions of appearance and how being marked as a person with disability changed how they interacted with society. Subsequently, many participants preferred to avoid living with this stigma, and even felt some degree of embarrassment acknowledging this reality.

Participant 6 (74 years old male): ‘If it was smaller, I could hide it under my hair…Because I’m quite vain. (Laughter)'

Other participants liked that a more obvious device might actually proudly draw attention to their condition, almost in an act or rebellion to societal norms.

Participant 3 (82 years old male): ‘I mean if you’re going to be deaf you’re going to be deaf, so you might just as well flag it up’.

For these participants a hearing device being obvious was a bonus, although the underlying sentiment and psychology behind these preferences was complex and varied between participants. Some participants liked the idea of sleek devices that they saw as modern or cool but nonetheless still wanted to minimise any awareness the wider public might have about their using such a device. Some participants perceived the obviousness of a larger or brasher device as fitting in with modern society's cosmetic values: achieving typicality or “normality” rather than actually standing out.

Participant 22 (70 years old male): ‘no different to people walking around with the dirty great earphones’

A few male participants were vociferous about what they thought was the wrong sort of device appearance, with comments that eluded to embarrassment or shame and gendered ideas about appropriate typical appearance, that may relate to societal pressures at the time they grew up.

Participant 20 (68 years old male): ‘…with the Alice band on…I felt a prat’.

Participant 16 (68 years old male): ‘there’s no way I would have worn that’.

Many participants regardless of gender spoke about their own perception of the way either they or the men in their life (or even men in general, in society) appeared to interact with hearing devices and other assistive technology for health in a dysfunctional way. For some participants their ideas of values and behaviours which uphold traditional masculinity and strength affected their own coping strategies. Some participants felt masculinity or male norms changed the way D/deaf people behaved with others and specifically their acceptance of the need or benefit of aids if appropriate.

Participant 5 (47 years old female): ‘…because they get embarrassed… someone… whose hearing is getting low…he won’t have hearing aids. There’s a lot of men that won’t’.

There was an underlying concept that a strong front or strength could be damaged by an acknowledgement of deafness. The theme developed as participants explained any imperfections or differences related to deafness might be seen as weakness or a cause to “lose face” or respect. For some male participants it led to frustration with any device they perceived as less than perfect. Imperfection in the performance of assistive technology devices therefore became a valid and justifiable reason for rejection. This rejection of imperfect technology may directly mirror their own experiences of rejection or reflect their own internalised ideas about how society had previously treated them. Many described wilfully choosing a life of relative social isolation rather than a confrontation with those frustrations in full view or judgement of society.

Responses regarding device aesthetics largely correlated to personal preference about the appearance of any such hearing device (namely whether aids should be obvious/celebrated or unobtrusive/remain discrete). Some participants approved of the fact that new bone conduction devices looked like a music player or headphones which would give a positive aesthetic impression of confidence.

Participant 10 (61 years old woman): ‘People are obviously curious and every day, you find… people are looking at you’

When male participants spoke about “feeling seen” like this, many male participants spoke about needing to demonstrate their strength. Displaying strength and power was described, first at school, then in the workplace and many felt this was tied intrinsically to initial perceptions and aesthetics, reflecting on their long-term use of hearing devices.

## Discussion

This study sought to explore the experiences and opinions of participants living with conductive deafness and hearing aid use throughout their lives. Most of the participants we spoke to experienced deafness from a young age. It is known that many children and young people who are deaf live in relative isolation from other people who are deaf and this is likely to make sharing, exploring and connecting over similar experiences more difficult (34). Previous studies have suggested that people with disabilities are largely absent from mainstream healthcare focused research (35) and as clinicians and researchers interested in intersections between hearing health and all other factors which can impact on health through the life course, we sought to explore these impacts in collaboration “with” rather than “about” these participants (34). Trends in research seem to suggest that issues of unpopularity or marginalisation amongst typically hearing peers are improving (to levels experienced by typically hearing youth) for young people in mainstream settings. However many children who are deaf still feel burdened by not wanting to attract unwanted attention and emphasise their felt need for “normalcy” (36). The aforementioned visibility of deafness seems to generate considerable stigmatisation. In our participants this stigma either appeared to decrease with age or else resilience and self-contentedness in the face of the stigma improved with age. It has been previously reported that contextual pressures around the time of identifying as D/deaf are different depending on age of onset (11). Children and younger adults sometimes benefit from developing their identities and participation in social groups at a time in which their hearing loss might be a source of connectedness within these formations. However, the recalled events of our participants suggest their experience was more akin to late-deafened adults who generally describe the significance of trying to maintain their “normal” identity before hearing loss (11).

We found that most participants described a challenging relationship with deafness, but that this relationship changed over time, with norms and acceptance changing both personally and with society. The three principle themes of the interviews alluded to the major sources of difficulty felt by our cohort. “The technological world” represented daily struggles living against elements of society both in childhood and with older approaches to medicine and health. The theme also developed from challenges when seeking to integrate with the help (and sometimes hindrance) of behavioural strategies and assistive or leisure technologies, particularly around entertainment involving music or television. “Normalised Marginalisation” highlighted that even in recent years, stigma and normalisation of typical needs and behaviours at school and in adulthood had devastating impacts on the self-perceived outcomes throughout the entire life-course of our participants. It remains to be seen in a younger cohort of participants with hearing loss whether societal norms in school has changed in recent years. Finally, “the Visible Display of D/deafness” was a theme unifying a battle between identify, typicality, notions of norms of vanity, norms of masculinity and traditionally acceptable aesthetics. These issues were raised by participants of all genders as damaging to acceptance and integration with society and use of assistive technology for many participants and their families.

People who are deaf or who prefer to identify as having hearing loss or hearing impairment, may experience dependence not just on technology but also on other people, something which can evoke feelings of precariousness and vulnerability (23). This has been described previously in older people dealing with this loss of power in relationships as a “rhythmical journey from despair to contentment, travelled over time” (37).

Currently hearing aids are used by only 76% of those who need them in high income settings (38) and as little as 3% in low income settings (39). An estimated 90% of people with disabling hearing loss live in low & middle income countries (40) and further reports of barriers to hearing aid adoption exist. When patients in lower resourced health systems do access hearing services, uptake of conventional hearing aids can be as low as 28% for adults and 15% in children with identified hearing issues respectively (41). Assessments could, and should, be made for alternative assistive technology for hearing by targeting areas where our participants identified the greatest difficulties, i.e., in studies where they are deployed in a non-healthcare setting e.g., schools and workplaces. In schools this could be through existing partnerships with Teachers of the Deaf. Participants in our cohort who overcame barriers described the significant transformative potential of such devices. In older adults, studies from the USA suggest health-seeking behaviours regarding hearing health are related to knowledge, attitudes, stigma, perceived competence and autonomy (42). Self-determination was a key factor in decision making about health seeking and device use in our participants. Future studies should asses how this relates to the hearing health and AT behaviour of children at the start of their journeys with hearing impairment and AT use.

We feel the responses and themes generated with the participants in this study could reasonably reflect experiences of similar patients across the UK whom also live with conductive hearing loss. We do not wish to make epidemiological inferences about our small cohort but hope this data will show that a broad mix of ages and genders was assessed. Several demographics were not assessed including children and adults over 82 years old. Additional limitations include that no D/deaf participants who communicate primarily with sign language were involved and none of the research team can communicate fluently in sign, which may have excluded valuable insights from alternative experiences of conductive deafness and engagement with society. No participants currently living in the North of the UK were interviewed due to the study location and budget (although some participants had been children in the North) but differential prevalence of hearing loss and associated regional lifestyle outcomes has suggested socioeconomic evidence of a North–South divide which may impact on countrywide interpretability of our findings (2). Further considerations regarding our conclusions include the positionality of the lead researcher (Male, surgeon, from South of UK with typical hearing) and the rest of the team (all typically hearing) which may have led to disproportionate emphasis or themes being developed which placed value on intervention.

Greater qualitative and quality of life analysis of D/deaf participants in all income settings and geographies are required if hearing health professionals seek to best support their health outcomes. Research in this area should seek validity and grounding in partnership with people living with conductive deafness. Our findings suggest that some behaviours and lifelong impacts are created from early childhood experiences of hearing impairment and school. Whilst some societal paradigms related to masculinity and aesthetics may have started to change across our age range of participants, concerns about avoidance of health seeking, behaviour related to isolation, control and response to bullying by childhood peers could still be present. These impacts altered behaviours, confidence, educational directions and job prospects for many of our participants. Future research should aim to further our ability to leverage economically as well as professionally and morally for greater investment in the lived experience of patients with hearing loss. We recommend that hearing health professionals and researchers should seek to explore primary school based interventions where national screening programmes do not exist, and liaise with education and teacher of the deaf services where health systems are better resourced. We hope to communicate these insights on experience and potential to benefit with hearing healthcare professionals, patient and public involvement groups, and local and national commissioners across the world.

This study provides a thematic analysis of semi-structured interviews with adults known to have conductive hearing loss in the UK. It has provided some insights into the spectrum of their lived experience as related to assistive technology for hearing. Many of these participants had an initially difficult relationship with their deafness, including how they felt and viewed themselves and how they interacted with society, particularly as children and young adults, but many described a positive change in their contentment and quality of life with the passing of time. The potential for benefit of assistive technology for hearing health was deemed by many participants to be a necessary bridge integrating them in relationships with other people in society, particularly in an increasingly technological-embracing modern world. It has been documented that for some individuals, where technology announces the hidden status as D/deaf, it can beneficially “manage societal expectations” and even support communication and self-expression (43).

For those who were able to describe their early experiences of deafness as school children, the struggle to engage with their education, their peers and society as a whole was a significant impact. These early notions of disability, typicality and social norms persisted into adulthood. Many of the participants talked about aesthetics, appearance and vanity, and tied notions of typicality and normality to strength, with some choosing to hide their deafness but others proudly embracing this difference. It was suggested that male participants in particular often perceived that deafness represented a degree of otherness or difference that was difficult for society to embrace, something that was complicated by traditional ideas of masculinity and traits that were seen as typically male, such as strength or confidence. Participants both young and old talked positively about their hopes for change in society and increasing opportunities for inclusivity. Many participants described their adoption of new technology or devices as “*transforming their life*” and their quality of life without the aids as significantly impaired. Adoption of certain types of technology will not be universally agreed or successful and care is required to prevent this from exacerbating health and social inequity and worsening the “digital divide”. These inequalities could be experienced particularly in contexts where assistive technology and healthcare may require increased health education awareness or out-of-pocket payments (44). In particular, when assistive technology for hearing are considered for school age children (and younger) it is important to remember there remains significant unexplained variability in performance and achievement both between D/deaf children and between D/deaf children and their typically hearing peers and any future work targeted at children should consider the wider intersections children may face (45). A key component of this will hopefully involve integration between multidisciplinary services and professionals across the healthcare and education spectrum, including audiologists, speech and language therapists, paediatricians, teachers, teachers of the Deaf, audio-vestibular physicians, ear nose and throat surgeons, health technologists and designers.

It is hoped that these insights from potential assistive technology users will help clinicians and researchers concerned with hearing health to continue to develop sensitive and acceptable solutions to improve people's lives. Providers of AT and researchers and clinicians for hearing health in general should seek greater collaboration and integration in the design and delivery of acceptable and effective devices with this population group. There is a need for wider conversations about interventions and services that are catered for the variety of experiences and expectations of individuals with hearing loss and D/deafness.

## Conclusion

This study considered the relationship that adults with conductive hearing loss can have with their deafness, with assistive technology to aid with their hearing, and with society in general. This included insights about how they felt and viewed themselves, and how ideas about typicality and aesthetics governed a number of their decisions and interactions both as children and young adults. The potential for benefit of assistive technology for hearing health was deemed by many participants to be a valuable bridge integrating them in relationships with other people in society, once they found an appropriate or personalised option that suited them. For many participants this took time. Early notions of disability, typicality and social norms experienced as children frequently persisted into adulthood. Ideas about appearance of hearing devices and specific potential of non-surgical bone conduction devices in this population with conductive deafness were suggested. The overarching theme of a changing relationship over time may indicate that future attempts to deliver improved uptake and adoption of hearing aids for individuals with conductive hearing loss will need to explore the themes mentioned here. In particular, will interventions be able to address underlying concerns about this “visual announcement of D/deafness”. We hope insights from these interviews will be considered by both patients and professionals making choices about provision of hearing health assistance for individuals with conductive hearing loss.

## Figures and Tables

**Table A1:** Demographic data (Age median and range, gender, numbers in each hearing threshold category).

Median Age in years (range)	70 (21–82)
Self-reported gender	
Male	15
Female	16
“Bi-gender” or “any”	2
No. Participants in each Hearing threshold category (dBA)	
>20–34	19
35–49	10
50+	4
Self-reported ethnicity (all phrases recorded verbatim as per participants request)	
White British	9
White	5
English	4
European	3
Irish	2
Scottish	1
Indian	1
mixed white	1
n/a	1
Normal	1
Oriental	1

## Data Availability

The anonymised raw data supporting the conclusions of this article will be made available by the authors, without undue reservation.

## Appendix 1

Demographics and summary statistics of ninterviewees

## Interview schedule

1. Could you tell me the story of your hearing?
  – Please describe how your hearing affects your life?
  – physical/emotional/behavioural/
  – communication/music/localisation
2. Could you tell me your opinion about the hearing aid you normally use?
3. Could you tell me what is most important to you about any hearing device?
  – family, friends, work, relationships?
4. What did you think of this new hearing device?
5. Why did it work well/badly?
6. If you had a friend who had to wait to have a hearing aid, what would you say to them about getting this device in the meantime?
7. From your perspective, what would you find most useful for us to change about the new device?
8. Do you have any other comments you would like to make about hearing devices?
9. Would you like us to send you a copy of the study?
